# Visual background information modulates motor contagions in humans

**DOI:** 10.1038/s41598-024-69535-9

**Published:** 2024-08-13

**Authors:** Hiroto Saito, Kentaro Fukuchi, Masahiko Inami, Gowrishankar Ganesh

**Affiliations:** 1 Research Center for Advanced Science and Technology, The University of Tokyo, 4-6-1 Komaba, Meguro-ku, Tokyo 153-8904 Japan; 2 School of Interdisciplinary Mathematical Science, Meiji University, 4-21-1 Nakano, Nakano-ku, Tokyo 164-8525 Japan; 3 UM-CNRS Laboratoire d’Informatique de Robotique et de Microelectronique de Montpellier (LIRMM), 161, Rue Ada, Montpellier, France

**Keywords:** Human behaviour, Sensorimotor processing, Motor control

## Abstract

Motor contagions refer to implicit effects induced by the observation of actions made by others on one’s own actions. A plethora of studies conducted over the last two decades have demonstrated that both observed and predicted actions can induce various kinds of motor contagions in a human observer. However, motor contagions have always been investigated with regard to different features of an observed action, and it remains unclear whether the background environment in which an observed action takes place modulates motor contagions as well. Here, we investigated participant movements in an empirical hand steering task during which the participants were required to move a cursor through a visual channel after being presented with videos of an actor performing the same task. We manipulated the congruency between the actions shown in the video and the background channels and examined whether and how they affected the participants’ own movements. We observed a clear interaction between the observed action and its background. The movement time of the participants’ actions tended to increase or decrease depending on whether they observed a faster or slower movement, respectively, and these changes were amplified if the background was not congruent with the action contained within it. These results suggest that background information can modulate motor contagions in humans.

## Introduction

Human motor behavior is implicitly influenced by the observations of actions performed by others; these effects are referred to as *motor contagions*^1,2^. Motor contagions demonstrate how our behaviors can be influenced by others near us and provide interesting insights into how the visual and motor systems in our brains interact. These effects have thus attracted much attention in sports science^3–8^ and neuroscience research^1,9–11^.

Most of the motor contagions investigated in the literature are so-called *action-imitative contagions* (AICs)^12^, which are induced simply by the observation of actions and have a signature characteristic: they cause certain features of a person’s action to become similar to that of the observed action. These features include kinematics^1,13,14^, outcome^3–5^, or overall behavior features^15^. In addition, the effect of a motor contagion is modulated by its context, such as the presence of a goal or an intention^16^ and social information^17^, as well as the features of the action itself. Alternatively, recent studies have described the presence of a second type of motor contagion, namely, *prediction error-induced contagions* (PECs), which are not determined only by the observed actions but also by what the observer predicts these actions to be^7,8,12,18^.

However, while all these studies have examined the effect of an observed motion on one’s own action, it remains unclear whether and how the observed visual background information, in which the action of the other person takes place, affects one’s behavior. Several recent studies reported that the congruency of visual context cues (whether the observed cues correspond to actions) presented during motion observations modulates the motor responses induced by the observed motion^19–21^. While the responses examined in these studies were explicit and intentional, these results led to our hypothesis that the congruency of visual background information may also influence motor contagions. This effect may have been missed until now because all previous motor contagion studies (those concerning both AICs and PECs) only studied action modulations within one static environment.

Here, to investigate whether and how visual background modulations affect motor contagions, we utilized an empirical hand *steering task*^22,23^. We recorded the cursor movements made by actors in two types of channels with different profiles: $\mathrm {C_{wide}}$ channels, which became wider along the direction of the movement, and $\mathrm {C_{nar}}$ channels, which became narrower along the direction of the movement. A $\mathrm {C_{wide}}$ channel is characterized by human movements that tend to accelerate across the length of the channel ($\mathrm {M_{wide}}$), while a $\mathrm {C_{nar}}$ channel is characterized by movements that tend to deaccelerate across the length of the channel. The participants were then shown scenarios in a 2 $\times$ 2 design in which the two movements ($\mathrm {M_{wide}}$ and $\mathrm {M_{nar}}$) made by the actors were mixed with the background, making the action and background either congruent or incongruent. We then examined how observing these congruent or incongruent scenarios affected participants when they completed their own steering tasks through a visual channel with a constant width ($\mathrm {C_{const}}$). In a second experiment, we also tested whether the observation of a channel alone affected the corresponding participant’s movements. Over the two experiments described above, we observed that visual backgrounds can modulate motor contagions in humans observing actions.

## Results

### Experiment-1

**Figure 1:**
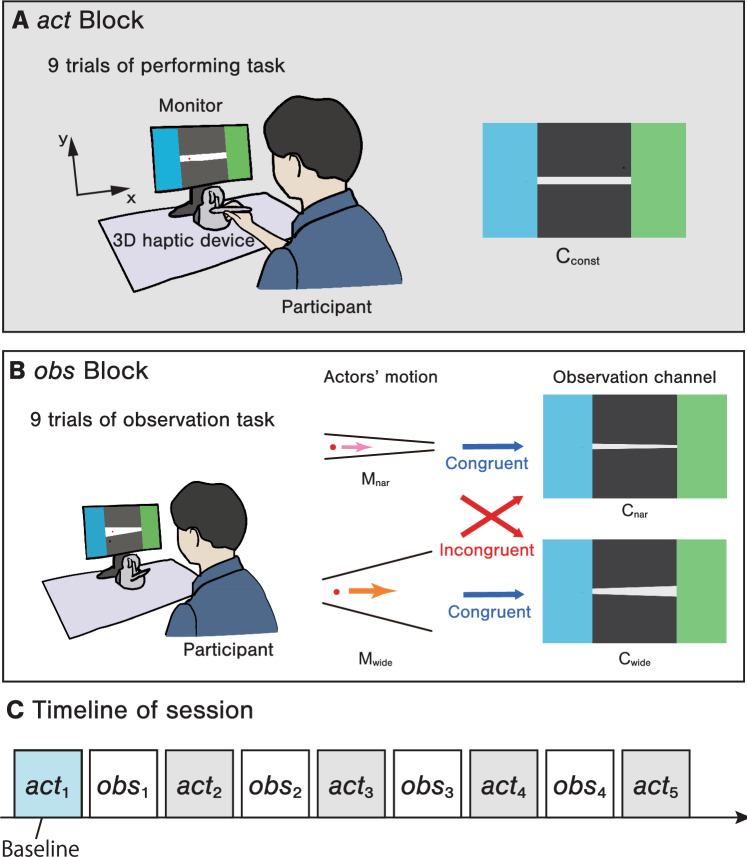
The experiments consisted of two types of blocks. (**A**) In an action block (*act* block), the participants performed a steering task in a straight channel ($\mathrm {C_{const}}$) as quickly as possible. (**B**) In an observation block (*obs* block), the participants watched videos of cursor motions implemented by unknown actors, who performed steering tasks with narrowing ($\mathrm {C_{nar}}$) or widening ($\mathrm {C_{wide}}$) channels. The combination of the actors’ motions and the channels in the observed video was controlled between sessions to create congruent and incongruent conditions (see Table 1). (**C**) Five *act* blocks were interspersed with four *obs* blocks in each session.

Twenty-four participants took part in Experiment-1. The participants performed five action blocks (hereafter *act* blocks; Fig. 1A) that were interspersed with four observation blocks (hereafter *obs* blocks; Fig. 1B) per session (Fig. 1C). In the *act* blocks, the participants were required to move a cursor through a straight channel with a constant width ($\mathrm {C_{const}}$; see Fig. 1 A right part) on a screen using a haptic stylus. The participants had to succeed in nine cursor movement trials consisting of three different constant widths (i.e., three times in each channel width), randomized in each block. Different channel widths were used to identify effects that were independent of specific participant movement velocities. In each trial, the participants were required to move through the task as quickly as possible without touching the walls. If the participant touched a wall while passing through the channel, the result was judged as a failed trial, and he/she had to repeat the task.

The participants took part in four sessions with identical *act* blocks. The order of the sessions was counterbalanced between the participants by randomly assigning each participant to one of twenty-four different permutation patterns (i.e., the factorial of four).

In the *Obs* blocks, the participants watched videos of cursor movements performed by unknown actors. During each session, they watched one of the two types of movements ($\mathrm {M_{wide}}$ or $\mathrm {M_{nar}}$) in one of the two types of channels ($\mathrm {C_{wide}}$ or $\mathrm {C_{nar}}$), as shown in Table 1 and 1 B (right part). This combination of channel and movement conditions was designed to create congruent and incongruent scenarios. A priori, we expected different motor contagions to be observed in conditions where the combinations of the channels and recorded motions were incongruent (i.e., $\mathrm {C_{nar}} \mathrm {M_{wide}}$ and $\mathrm {C_{wide}} \mathrm {M_{nar}}$) than those produced when the channels and recorded motions were congruent (i.e., $\mathrm {C_{nar}} \mathrm {M_{nar}}$ and $\mathrm {C_{wide}} \mathrm {M_{wide}}$).

Each trial involving an *obs* block started with the presentation of a fixation point in the center of the monitor. The participants were asked to maintain their gaze on this point until the recorded cursor movement video began. The participants were required to keep their eyes on the cursor during the video presentation.

**Figure 2:**
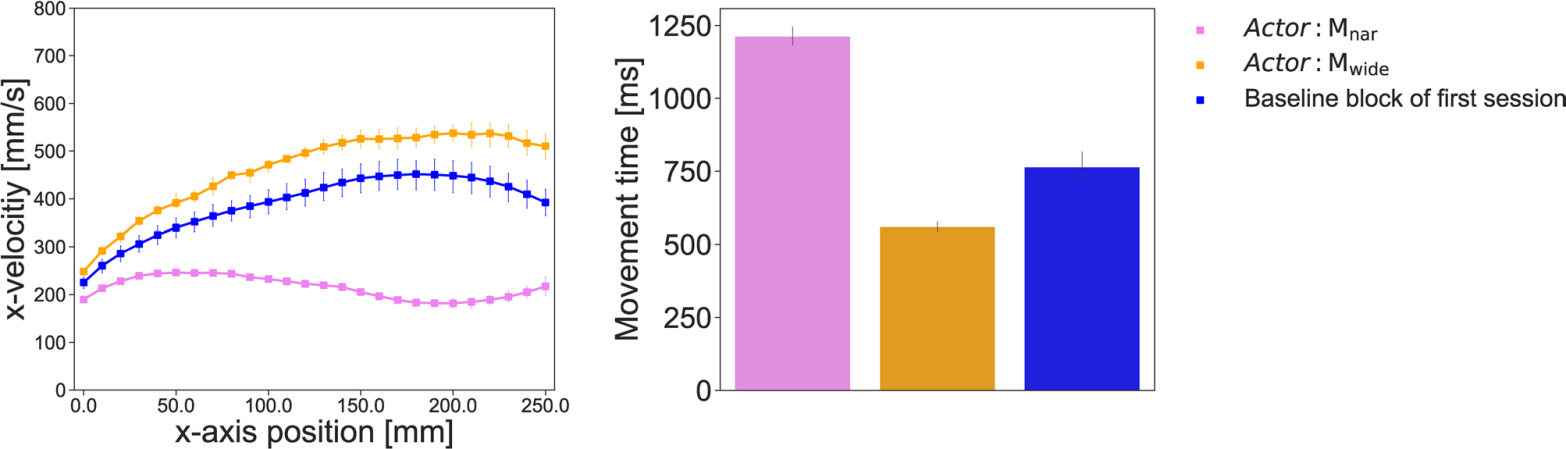
The x-velocity of the movement of each actor in each *obs* block condition (i.e., $\mathrm {M_{wide}}$ and $\mathrm {M_{nar}}$) and the first *act* block of each participant during the first session (i.e., the movements before the observation period) in Experiment-1. The right panel shows the average velocity values. The left panel shows the velocity observed during the channel passing task at every 10 mm in the x-direction of the channels. The error bars show the standard errors.

First, we tested whether the velocity of $\mathrm {M_{wide}}$ was higher than that of the participants’ initial *act* block and whether the velocity of $\mathrm {M_{nar}}$ was lower. $\mathrm {M_{wide}}$ tended to accelerate gradually during the process of traversing the channel, while $\mathrm {M_{nar}}$ did not exhibit much of a velocity change but tended to decelerate slightly in the middle of the channel. The participants’ movements in the initial session also tended to accelerate gradually but not as much as those of $\mathrm {M_{wide}}$ (Fig. 2). The average differences between the maximum velocity and the velocity at the starting line were $310\,(s.d. = \pm {53})\,{\hbox {mm/s}}$ for $\mathrm {M_{wide}}$, $79\,(s.d. = \pm {27})\,{\hbox {mm/s}}$ for $\mathrm {M_{nar}}$, and $257\,(s.d. = \pm {133})\,{\hbox {mm/s}}$ for the participants’ first *act* block (9 trials $\times$ 24 participants = 216 trials in total). In contrast, the average *movement time* (MT), the time required to pass through a channel in each trial, was $560\,(s.d. = \pm {51})\,{\hbox {ms}}$ for $\mathrm {M_{wide}}$, $1214\,(s.d. = \pm {171})\,{\hbox {ms}}$ for $\mathrm {M_{nar}}$, and $765\,(s.d. = \pm {312})\,{\hbox {ms}}$ for the participants’ first *act* block. Overall, the average $\mathrm {M_{wide}}$ was faster (i.e., higher velocity / lower MT) than the participants’ own initial sessions, while $\mathrm {M_{nar}}$ was slower (i.e., lower velocity / higher MT) than the participants’ initial sessions. The accelerations observed while the cursor passed through the channels also produced a similar trend.

**Table 1:** Experiment-1 consisted of four sessions that presented participants two movement conditions ($\mathrm {M_{wide}}$ and $\mathrm {M_{nar}}$) mixed with the two channel shapes ($\mathrm {C_{wide}}$ and $\mathrm {C_{nar}}$) that they were shown in. Under the congruent conditions, the presented actor movements were derived from the same channel as that in which they were presented ($\mathrm {C_{nar}} \mathrm {M_{nar}}$ and $\mathrm {C_{wide}} \mathrm {M_{wide}}$); the incongruent conditions presented actor movements extract from a different channel than that in which they were presented ( $\mathrm {C_{nar}} \mathrm {M_{wide}}$ and $\mathrm {C_{wide}} \mathrm {M_{nar}}$). Note that the participants always performed their tasks in straight channels.

	Congruency	
Movements	Congruent	Incongruent
Movement in a Narrowing Channel ($\mathrm {M_{nar}}$)	$\mathrm {C_{nar}} \mathrm {M_{nar}}$	$\mathrm {C_{wide}} \mathrm {M_{nar}}$
Movement in a Widening Channel ($\mathrm {M_{wide}}$)	$\mathrm {C_{wide}} \mathrm {M_{wide}}$	$\mathrm {C_{nar}} \mathrm {M_{wide}}$

Next, we analyzed the effects of an observation on the participant’s own task performance in the *act* blocks using a two-way (2 *congruency* types $\times$ 2 *movements*) repeated-measures analysis of variance (ANOVA). The task performance of a participant was evaluated using the *error rate* (the ratio of trials in which they touched the channel wall in a block), the *peak distance* (the maximum vertical distance of the pointer from the center of the channel), and the MT. The *error rate* is the ratio of the number of failed trials to the total number of block trials required to succeed in nine trials. Apart from the *error rate*, we measured the *peak distance* to determine the participants’ motion accuracy variations during successful trials, as well as their movement times (MTs), i.e., the times required to traverse the channel.

The first *act* block was performed before the observation period as a warm-up for the participants. We observed that the participant behaviors were similar across the first block of each condition for the above three measures (a one-way ANOVA conducted across the four combination conditions did not show significant differences; *error rate*: $F(3,23) = 2.345, p = 0.080, \eta ^2_P = 0.093$; *peak distance*: $F(3,23) = 0.922, p = 0.435, \eta ^2_P = 0.039$; MT: $F(3,23) = 1.724, p = 0.170, \eta ^2_P = 0.070$). We therefore used the average of these first blocks in each condition, calculated for each participant, as the baseline. During the two-way ANOVA, we used the differences between the baseline and the average values produced across *act* blocks 2 to 5, which were performed after the *obs* blocks, for the session data. The participants completed 4698 trials in total (including 378 failed trials); 947 trials (including 83 failed trials) involving the first *act* block were used as the baseline, and thus, a total of 3751 trials (including 295 failed trials) were used as performance data after completing the *obs* blocks.

**Figure 3:**
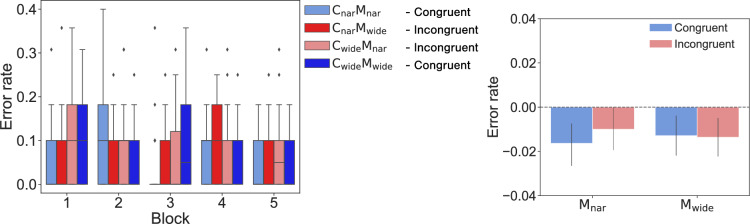
The *error rates* attained in Experiment-1. The left panel shows the *error rate* produced for each *act* block under each condition. The right panel shows the differences between the baseline and the average values obtained across blocks 2 to 5 under each condition. The error bars show the standard errors.

**Figure 4:**
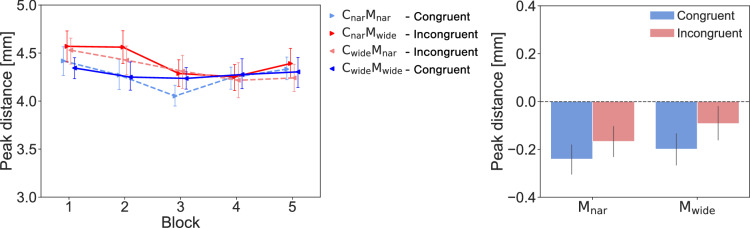
The *peak distances* attained during Experiment-1. The left panel shows the *peak distance* changes induced during the participants steering tasks conducted across the *act* blocks under each condition. The right panel shows the differences between the baseline and the average peak values attained across blocks 2 to 5 under each condition. The error bars show the standard errors.

No significant differences were observed across the *congruency* and *movements* conditions in terms of the *error rate* (Fig. 3). Normality was not rejected under any of the data groups, and a two-way repeated-measures ANOVA ($\alpha = 0.05$) did not show either main effects or interactions (*congruency*: $F(1,23) = 0.312, p = 0.582, \eta ^2_P = 0.013$; *movements*: $F(1,23)< 0.001, p = 0.995, \eta ^2_P < 0.001$; interaction: $F(1,23) = 0.126, p = 0.725, \eta ^2_P = 0.006$). Similarly, no significant differences were observed in the *peak distance* results (Fig. 4). Because normality was rejected for some of the data groups, we conducted a two-way repeated-measures ANOVA ($\alpha = 0.05$) after carrying out the nonparametric aligned rank transform (ART) procedure^24^. The results did not show either main effects or interactions (*congruency*: $F(1,23) = 0.802, p = 0.380, \eta ^2_P = 0.034$; *movements*: $F(1,23) = 2.208, p = 0.151, \eta ^2_P = 0.088$; interaction: $F(1,23) = 0.007, p = 0.932, \eta ^2_P < 0.001$). Overall, these results suggested that the differences between the observed movements and the predictions made about them did not show an effect on the accuracy of the participants’ motions.

**Figure 5:**
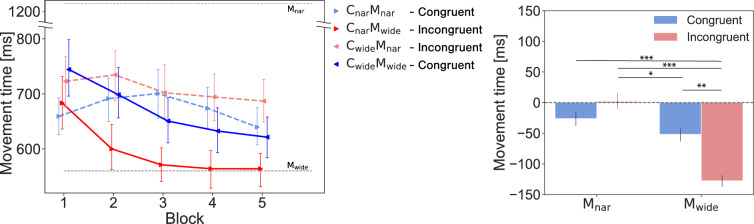
The MTs yielded in Experiment-1. The left panel shows the changes exhibited by the participants’ MTs across the *act* blocks under each condition. The right panel shows the differences between the baseline and the average values produced across blocks 2 to 5 under each condition. The error bars show the standard errors. $^*{} p < 0.05$, $^*{}^*{} p < 0.01$, $^*{}^*{}^*{} p < 0.001$.

However, the MT was found to be modulated by the *congruency* and observed *movements* conditions (see Fig. 5 left). We performed another two-way ANOVA with the ART, which showed a significant main effect concerning *movements* ($F(1,23) = 43.540, p < 0.001, \eta ^2_P = 0.654$) and a significant interaction ($F(1,23) = 11.260, p = 0.003, \eta ^2_P = 0.329$). The main effect of *congruency* lacked statistical significance ($F(1,23) = 2.848, p = 0.105, \eta ^2_P = 0.110$). A post-hoc interaction analysis, which included multiple comparisons among the six pairs consisting of four combinations, showed statistically significant differences among the average MTs of various pairs, as shown in Fig. 5 right): $\mathrm {C_{wide}} \mathrm {M_{nar}}$ > $\mathrm {C_{wide}} \mathrm {M_{wide}}$ ($adj.p = 0.037$); $\mathrm {C_{wide}} \mathrm {M_{nar}}$ > $\mathrm {C_{nar}} \mathrm {M_{wide}}$ ($adj.p <.001$); $\mathrm {C_{wide}} \mathrm {M_{wide}}$ > $\mathrm {C_{nar}} \mathrm {M_{wide}}$ ($adj.p = 0.001$); and $\mathrm {C_{nar}} \mathrm {M_{nar}}$ > $\mathrm {C_{nar}} \mathrm {M_{wide}}$ ($adj.p < 0.001$). Therefore, even when multiple participants observed the same movement, the effects of this result on the MT differed depending on the given visual background information, and the MTs were significantly shorter in the $\mathrm {C_{nar}} \mathrm {M_{wide}}$ condition than in the other conditions.

The above results suggest that while the accuracy (i.e., the *error rate* and *peak distance*) remained unaffected, motor contagions were induced in one’s MT by observing others’ movements. This effect may have been a result of the interaction between the observed action and the expected action (based on visual background information). Specifically, observing faster movements had the effect of accelerating participants’ movements, and this effect was more pronounced in the conditions, where the participants observed movements within an incongruent channel. Interestingly, an ANOVA conducted across the 2 *movements* $\times$ 2 *channels* conditions showed main effects for both factors (*movements*: $F(1,23) = 43.540, p < 0.001, \eta ^2_P = 0.654$; *channels*: $F(1,23) = 11.260, p = 0.003, \eta ^2_P = 0.329$; and no interaction: $F(1,23) = 2.848, p = 0.105, \eta ^2_P = 0.110$). This made us wonder whether observing a channel itself could affect participants’ movements independently of the observations of the movements. To confirm this hypothesis, we conducted Experiment-2.

### Experiment-2

**Figure 6:**
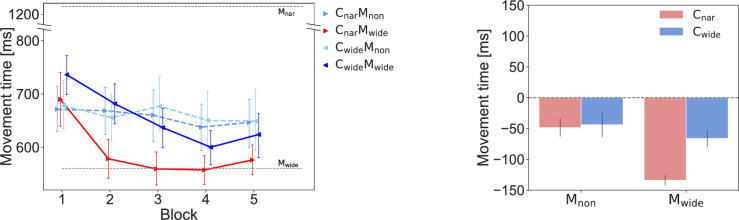
The MTs yielded in Experiment-2. The left panel shows the changes exhibited by the participants’ MTs across the *act* blocks under each condition. The right panel shows the differences between the baseline and the average values produced across blocks 2 to 5 under each condition. The error bars show the standard errors. Note that the main effect of the *presence of movement* was significant according to a 2-way ANOVA ($p = 0.046$).

To test whether the effects observed on MT in Experiment-1 could also be caused by channel observation alone as well as to examine the effect of observing faster movements compared to not observing movements, twelve participants who had prior experience from Experiment-1 participated in the additional Experiment-2. Although a sample size of N = 11 was proven sufficient based on the F-value observed in Experiment-1 when using the bias and uncertainty-corrected sample size tool developed by Anderson et al.^25^, we selected N = 12 as a more conservative choice. The procedures followed in Experiment-2 were the same as those used in Experiment-1, except for the fact that the participants observed only the $\mathrm {C_{nar}}$ and $\mathrm {C_{wide}}$ channels in each *obs* block trial and not the cursor movements within them (hereafter $\mathrm {C_{nar}} \mathrm {M_{non}}$ and $\mathrm {C_{wide}} \mathrm {M_{non}}$). The participants attended just two sessions involving the same *channels* (i.e., one each for $\mathrm {C_{nar}}$ and $\mathrm {C_{wide}}$). The orders of the sessions were counterbalanced among the participants.

Experiment-2 required the participants to perform the task in the $\mathrm {C_{const}}$ channel after observing either the narrow channel without any cursor movement ($\mathrm {C_{nar}} \mathrm {M_{non}}$) or the wide channel without any cursor movement ($\mathrm {C_{wide}} \mathrm {M_{non}}$). The output data were compared with the behaviors of the same participants in Experiment-1 after implementing a congruent observation ($\mathrm {C_{wide}} \mathrm {M_{wide}}$)) or an incongruent observation ($\mathrm {C_{nar}} \mathrm {M_{wide}}$) in the wide channel, as these settings had the greatest effects on the participants’ own motions in Experiment-1. We analyzed the effects of observing a channel using a two-way (2 *channels* $\times$ 2*presence of movement* conditions) repeated-measures ANOVA. We expected to see the main effect of the *channels* in the ANOVA if observing the channel profile affected the observer’s own steering task. On the other hand, we expected to observe the main effect of the *presence of movement* or an interaction if the cursor movement in the channel was required to produce contagion in the observer’s steering task. The other analysis protocols were the same as those employed in Experiment-1.

A total of 3485 trials (699 trials of baseline performance data, including 51 failed trials; 2786 trials of performance data obtained after the *obs* blocks, including 194 failed trials) were analyzed in Experiment-2.

We found that the MT was modulated only when cursor movements appeared (Fig. 6). Normality was not rejected, and a two-way ANOVA showed a significant main effect for the *presence of movement* ($F(1,11) = 5.077, p = 0.046, \eta ^2_P = 0.316$). The main effects of the *channels* ($F(1,11) = 2.263, p = 0.161, \eta ^2_P = 0.171$) and interactions ($F(1,11) = 3.783, p = 0.078, \eta ^2_P = 0.256$) were not significant. Observing faster movement had the effect of increasing the speed of the participant’s own movement, even when compared to the condition where movement was absent. On the other hand, the main effect of the channel observed in Experimen-1 was not observed in Experiment-2, which included the condition of no cursor movements within the channels. These results suggest that the differences in MT between channel conditions observed in Experiment-1 were not an effect of observing the channel itself, but an interaction between the cursor movement within it and the consistency of the channel.

## Discussion

In the two experiments conducted above, we explored whether and how the visual background information of an action modulates an observer’s motion. In Experiment-1, we observed that the MTs of the observers were influenced by an interaction between the movement of the cursor they observed and the background information (congruency with channel shape). In Experiment-2, we showed that observing the visual background itself did not affect the observer’s motion. Overall, the results suggest that background information modulates the motor contagion effect caused by observing the movement of a cursor.

We observed a significant MT interaction between the *movement* and *congruency* factors, and in the presence of incongruency, the AIC effect tended to increase. This result shows that background information modulate motor contagions. Several reasons could have contributed to this effect. First, the channel shapes may have created a visual illusion and modified the perceived cursor speeds within the channels. Specifically, the $\mathrm {C_{nar}}$ channel may have led to a general increase in the perceived speed of the cursor, leading to an increase in each observer’s own steering speed after observing movement in $\mathrm {C_{nar}}$ (Fig. 5). Similarly, the $\mathrm {C_{wide}}$ channel may have led to an overall decrease in the perceived cursor speed, leading to a decrease in each observer’s own steering speed after observing the movement in $\mathrm {C_{wide}}$. Second, the background may have modulated the competitive motivations of the observers, making them copy the performance of the actors. That is, observing an accelerated movement ($\mathrm {M_{wide}}$), which was typical of the $\mathrm {C_{wide}}$ channel, in the $\mathrm {C_{nar}}$ channel could have motivated the participants to quicken their own movements. Conversely, observing a deaccelerated movement ($\mathrm {M_{nar}}$) in the $\mathrm {C_{wide}}$ channel could have induced the participants to slow their own movements. This possibility is supported by the predictive coding model of social perception^26^ and our index of performance (IP), which is a generalized performance index that is independent of the given path conditions^27–30^. According to the predictive coding model, the participants could have inferred the actor’s intentions by observing their movements. We observed a strong correlation between the IP of the observed actions and the IP of the participants (see Fig. S1 in the Supplementary Information for more details). However, given that few data points were available for this analysis, further studies are required to confirm performance copying as the cause of the observed results. Finally, previous studies have reported that unexpected visual stimuli increase human attention^31,32^, so it is possible that attention changes may have contributed to the increased AICs. Further studies are needed to clarify whether one or more of these factors contributed to our observations.

We note here that we did not observe any PECs, which were observed in previous studies^7,8,12,18^. Prior to the experiments, we expected the PECs to manifest as acceleration changes opposing the observations according to the findings of previous studies^7,8,12^. That is, we expected the participants to decrease their own movement speeds when they observed accelerated movements in the $\mathrm {C_{nar}}$ channels (because they would expect a deaccelerated movement in this channel) and conversely to increase their movement speeds after watching deaccelerated movements in the $\mathrm {C_{wide}}$ channels. Some experimental design differences may explain the lack of PECs observed in this study. Specifically, PECs are known to be heavily modulated by the perceived goal of the actor^12^, and the participants in this study were not instructed in detail about the intentions or goals of the actors because we focused on environmental effects rather than the task instructions. Moreover, the participants’ own movements were presented on the screen in real time, and the presence of visual feedback in addition to AICs and environment-related contagions may have hidden any PECs induced in the participants’ behaviors. Furthermore, the observed video did not include information about the actors’ kinematics because this study focused on observing the channels and the movements of the cursor within them, and it is possible that kinematic observations are the key to the introduction of PECs. Finally, an interesting possibility is that PECs might be specific to the time at which the observed background is similar to one’s own task environment, while AICs are modulated more by the observed velocity rather than the background. Further studies are required to clarify these points.

In our experiments, the MT changes persisted despite the fact that the participants had visual feedback for their cursor. However, it remains unclear if the participants perceived these MT changes in their movements. The answer to this question is interesting with respect to the sense of agency (or the subjective perception of controlling one’s action) perceived by the participants towards their movements^33–37^ when suffering from motor contagions. Several previous studies have suggested the sense of agency to be modulated by the prediction errors generated by the internal models used for our movements^38,39^, and evaluating the effects of motor contagions on the sense of agency may be a way to quantify the effects induced by motor contagions on the learned internal models that drive one’s movements.

The finding that the underlying environment can affect motor contagions is novel and important for the design of systems that support training through observational motor learning^40,41^ and for implicitly driving users’ movements by using apparent motion^42^. A unique feature of our experimental results is that the $\mathrm {C_{nar}} \mathrm {M_{wide}}$ condition modulated the movements in a direction that yielded improved task performance (like Vasalya et al.^43^). The average MT was significantly shorter than that of the $\mathrm {C_{wide}} \mathrm {M_{wide}}$ condition, in which the observed motion itself was the same. In contrast, the error rate and peak distance were not affected by the background information during the observation. On the other hand, it is unclear how long these effects lasted. We hope to clarify this issue through further studies.

## Methods

### Task and apparatus

Our participants were required to pass a red cursor (2 mm in diameter) through the light gray channel and then move it from the blue area on the left to the green area on the right. The length of the channel was fixed at 250 mm throughout the experiment. The width of the channel changed in each trial (see the next subsection for more details). The participants were required to pass the cursor as quickly as possible without exceeding the upper and lower boundaries.

The participants sat facing an LCD monitor (Dell, 240 Hz, 24.5 inches) that presented visual information. They held the stylus of a 3D haptic device (3D Systems Touch, which is capable of 3D pointing with a ~0.055-mm nominal position resolution) with their dominant hand. Programmed forces restricted the movement of the haptic stylus in a plane parallel to the computer screen such that the participants could manipulate the cursor on the screen by moving the stylus in a plane parallel to the computer screen. The cursor movement shown on the screen was scaled to match the physical movement of the stylus. The participants’ movements were recorded by the encoder of the pointing device at a sampling rate of 1000 Hz. The latency of the real-time visual feedback was 17–21 ms based on a simultaneous 240-fps video recording of the hand motions and visual feedback presented on the screen using an external camera.

### Participants

#### Experiment-1

Twenty-four naïve participants (17 males and 7 females aged 19–36, mean age: 24.4 years, standard deviation: 4.19 years) took part in Experiment-1. This sample size was determined based on that calculated using G * Power 3.1 with a repeated-measures within-factors ANOVA ($$\alpha = 0.05$$, $$\beta = 0.80$$, $$f =0.25$$, number of measurements = 4, correlation among repeated measures $$ = 0.5$$, nonsphericity correction $$ = 1$$^44^). All the participants had normal or corrected-to-normal vision and no disabilities. All the experiments were approved by the Life Science Research Ethics and Safety Office at the University of Tokyo, Japan (approval number: 19-414). All the experimental procedures described below were conducted in accordance with the guidelines of the Declaration of Helsinki^45^ and the procedure approved by the above ethics committee. The participants signed an informed consent form before taking part in the experiment and received compensation of JPY 2,100 after completing the work.

#### Experiment-2

Twelve participants (9 males and 3 females aged 20–31, mean age: 24.3 years, standard deviation: 4.03 years) took part in Experiment-2. This sample size was determined based on the main effect of movement on the MTs in Experiment-1 using the bias and uncertainty-corrected sample size tool developed by Anderson et al.^25^. A sample size of N = 11 has proven sufficient for detecting the effect with a statistical power level of $\beta = 0.80$, an assurance level of 0.90, and a significance level of $\alpha = 0.05$. We determined our sample size of N = 12 as a more conservative choice and to counterbalance the task orders between subjects. Also, this sample size corresponds to an effect size of $f = 0.40$ using G*Power 3.1, and the effect size for the ANOVA where significant differences were found in Experiment-1 was larger than this. To compare the results of Experiment-1 and Experiment-2, we recruited participants who all had experience with Experiment-1. Experiment-2 was conducted on the same day as Experiment-1.

### Experimental design in Experiment-1

In an experimental session, five *act* blocks (Fig. 1A) were alternately interspersed with four *obs* blocks (Fig. 1B), as shown in Fig. 1C. In the *act* blocks, the participants performed the steering task themselves; in the *obs* blocks, they observed the recorded images of the steering task being performed by unknown actors. We investigated the effect of the interaction between motion the observation and the environment observation (i.e., the channel shape) on the performance of each participant. Each participant took part in a session of the experiment under each of these conditions.

The observation conditions were set by combining the *channels* ($\mathrm {C_{nar}}$ and $\mathrm {C_{wide}}$) and the *movements* of the cursor ($\mathrm {M_{nar}}$ and $\mathrm {M_{wide}}$) presented in the *obs* blocks of each session.

In the *obs* blocks, the following two types of channel conditions were employed for the observation task, where the channel width changed linearly from the starting line (i.e., the boundary with the blue area on the left) to the endpoint (i.e., the boundary with the green area on the right); see Fig. 1B).

- $\mathrm {C_{nar}}$: The channel width at the starting line was 16 mm, and the width at the end line was 8 mm (i.e., the channel width at the end line was half of the that at the starting line).
- $\mathrm {C_{wide}}$: The channel width of the starting line was 16 mm, and the width of the end line was 32 mm (i.e., the channel width at the end line was twice that at the start line).

For the two types of observed movements, the following types of data were recorded for the actor’s cursor motion.

- $\mathrm {M_{nar}}$: The data recorded when the actor passed the cursor through $\mathrm {C_{nar}}$.
- $\mathrm {M_{wide}}$: The data recorded when the actor passed the cursor through $\mathrm {C_{wide}}$.

The observation conditions were a combination of the two *congruency* $\times$ two *movements* settings. The *congruency* conditions were decided based on whether the cursor movements were presented in the same channel as that used to record the actor. In other words, $\mathrm {C_{nar} M_{nar}}$ and $\mathrm {C_{wide} M_{wide}}$ were categorized as belonging to the *congruent* condition because the cursor movements were presented in the same channel as that used to record the actor. On the contrary, $\mathrm {C_{wide} M_{nar}}$ and $\mathrm {C_{nar} M_{wide}}$ were categorized as belonging to the *incongruent* condition because the cursor movements were presented in a different channel from that used to record the actor.

In the *act* blocks, the channel width was constant from the starting point to the endpoint, as shown in Fig. 1A. The channel width was selected from the following three conditions by a pseudo-random algorithm in each trial: 12 mm, 16 mm, and 20 mm.

### Procedure of Experiment-1

Before starting the main sessions, the participants took part in training sessions. After that, they participated in four sessions consisting of each of the above mentioned conditions. The orders of the sessions were counterbalanced among the participants. Each session involved five *act* blocks and four *obs* blocks. The participants had a break of at least three minutes between the sessions.

#### Training sessions

The participants took part in three types of training sessions to practice performing the steering task. In the first training session, they started with a wide $\mathrm {C_{const}}$ channel (24 mm) , and as they became used to the operation, they experienced tasks with narrower $\mathrm {C_{const}}$ channels (12 mm at the end of the session). In this session, the participants were able to confirm the operations and their comfortable postures to become sufficiently familiar with the task. In the second training session, the participants experienced tasks with $\mathrm {C_{wide}}$ and $\mathrm {C_{nar}}$. We conducted this session to give participants an experiential understanding of the difficulty of the tasks involving each channel with respect to the observation process. In the final training session, the participants experienced the tasks under the three conditions of $\mathrm {C_{const}}$ in an order determined by a pseudo-random number, as with the *act* blocks. This session included nine trials (three trials for each $\mathrm {C_{const}}$).

#### *Act* blocks

In the *act* blocks, all participants were required to perform the steering task as quickly as possible and with as few failures as possible. Before a trial began, the $\mathrm {C_{const}}$ of this trial and the cursor were displayed. Furthermore, the stylus was fixed to the starting position in the blue area. Then, each participant started the trial by pressing the button on the stylus with their thumb. During the trial, the stylus could be moved freely in the upward, downward, left, and right directions. The participant manipulated their stylus to make the cursor pass through the channel by holding down the button. A clamping force from the haptic device helped to restrict the participant movements in a plane parallel to the monitor. When the cursor reached the green area, a sound was presented to indicate success. Then, when the participant released the button, the stylus was locked in its current position. If the participant touched the upper or lower border while passing through the channel, a sound indicating failure was presented, and the stylus was fixed in that position. Then, the display blacked out and the stylus automatically returned to the initial position. When the stylus reached its initial position, it was fixed, and the channel and cursor of the next trial were displayed.

The participants performed nine trials of the *steering task* in each block. If a participant touched the upper or lower boundary while passing through the channel, the result was judged as a failed trial and was not counted, and a trial with the same channel width condition was added to the end of the block. In other words, the participant had to succeed in the steering task nine times (i.e., three times for each channel width) per block.

#### *Obs* blocks

In the *obs* blocks, the participants were required to observe the cursor motions performed by the actors. In each *obs* block, nine samples were randomly selected out of 30 samples (3 actors × 10 samples) of motion data for the *movement* condition in that session (i.e., $\mathrm {M_{wide}}$ or $\mathrm {M_{nar}}$), and each sample was shown once. Before the start of the first *obs* block, the participants were informed that the video of the steering task was performed by others under a channel (i.e., $\mathrm {C_{wide}}$ or $\mathrm {C_{nar}}$), and each video was shown nine times. When a trial started, a black background and a cross-shaped gazing point at the center of the screen were shown for 1000 ms. Following this, the gazing point disappeared, a channel and a cursor appeared, and the cursor moved from left to right. After the cursor reached the area on the right, the screen blacked out, and the next trial started after a 1000 ms delay. The participants were required to keep their eyes on the gazing point at the start of the trial and on the cursors passing through the channel during the trial.

#### *Obs* block videos

To create video images for the *obs* blocks, the two-dimensional coordinates of the cursor manipulated by the actor were recorded. The three actors were given the same basic task instructions as those presented to the participants and repeatedly performed steering tasks in the $\mathrm {C_{wide}}$ and $\mathrm {C_{nar}}$ conditions. The coordinate data were recorded at a sampling frequency of 240 Hz in each trial using the same equipment as that employed in the experiments. The recording process for each actor continued in the trials until 10 samples per channel condition were collected. We collected only the motion data that fell within the $\mathrm {C_{nar}}$) condition for the $\mathrm {M_{wide}}$ condition. This was because the main experiment had a condition in which the $\mathrm {M_{wide}}$ condition was presented in the $\mathrm {C_{nar}}$) condition (i.e., $\mathrm {C_{nar} M_{wide}}$). In other words, for the trials in the $\mathrm {C_{wide}}$ condition, even if they were successful, the data were not collected if they did not fall within the $\mathrm {C_{nar}}$ condition. However, during the recording process, the actors were not informed whether the data had been collected. Therefore, the motion speed was not intentionally suppressed to keep the actors within the $\mathrm {C_{nar}}$ condition.

### Experimental design and procedure of Experiment-2

In Experiment-2, the participants observed only the channels without the moving cursors in the *obs* blocks. The two channel conditions were the same as those utilized in Experiment-1 (i.e., $\mathrm {C_{nar}}$, $\mathrm {C_{wide}}$). The participants took part in sessions for each of the conditions. The session consisted of five *act* blocks and four *obs* blocks, as in Experiment-1. The orders of the sessions were counterbalanced among the participants.

In each *obs* block, the following trial was conducted nine times.

- A black background with a cross-shaped gazing point placed at the center of the screen was first presented for 1000 ms.
- Then, the gazing point disappeared, and the image of the channel ($\mathrm {C_{nar}}$ or $\mathrm {C_{wide}}$) was presented for 1500 ms.
- The screen was finally blacked out, and blank screen was presented for 1000 ms, i.e., until the next trial.

The participants were required to keep their eyes on the gazing point at the start of the trial and on the channel during the trial. All the other task designs and procedures were the same as those implemented in Experiment-1.

### Data analysis

The error rate and maximum vertical peak distance metrics were measured as task accuracy indicators. The error rate was calculated as the number of failed trials divided by the total number of trials in each block. The peak distance was the maximum vertical offset of the cursor trajectory from the center of the channel during each trial. Only successful trials were included in the peak distance analysis; unsuccessful trials were excluded. The trajectory of the cursor from the starting line to the end line was recorded using the encoder of the input device (with a position resolution of   0.055 mm) at a refresh rate of 1000 Hz. Among the cursor position samples recorded for each trial, the farthest vertical distance from the center line was defined as the peak distance. The time elapsed from passing the starting line to passing the end line in a successful trial was defined as the ‘movement time’ (MT).

In Experiment-1, statistical analyses were performed to determine the differences between the baseline (the average value of the first *act* block between two sessions) and the average values of *act* blocks 2–5 for the three parameters listed above (i.e., the error rate, peak distance, and MT). We analyzed the effects of the observation process on a participant’s task performance in each of the *act* blocks using a two-way (2 *congruency* conditions $\times$ 2 *movements*) repeated-measures ANOVA. In Experiment-2, we conducted a two-way (2 *channels* $\times$ 2 *presence of movement* conditions) repeated-measures ANOVA on MT by using the observed $\mathrm {M_{non}}$ data that were newly acquired in Experiment-2 and the observed $\mathrm {M_{wide}}$ data from Experiment-1. Before implementing the ANOVA, we confirmed the normality of the data using the Shapiro-Wilk test. If normality was rejected, we conducted the ANOVA using the ART procedure^24,46^. A post-hoc analysis (multiple comparisons) was conducted using a pairwise t-test for the normal data, and the ART procedure was employed to conduct multifactor contrast tests^47^ for the non-normal data. The p-values were adjusted using Holm’s sequential Bonferroni procedure^48^.

### Steering task

**Figure 7:**
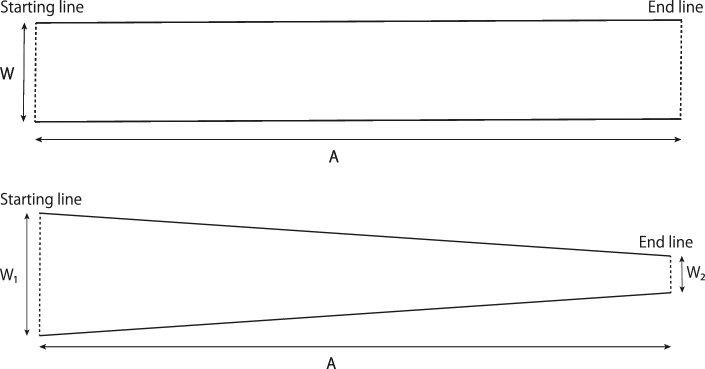
The straight channel (upper image) and narrowing channel (lower image) used in the steering task^22^.

To examine the relationship between the environment and movement with respect to the motor contagions, we employed a behavioral task called the *steering task* proposed by Accot and Zhai^22,23,28^. In this task, users manipulate a visual object such as a mouse cursor to pass through a narrow path. In this steering task, the so called *steering law model*^22,23,28^ defines the performance of the users. This *steering law model* was derived from *Fitts’ law*^49^ and has been confirmed to fit various conditions and GUI operations such as indirect-input styluses^22^, mice, touchpads, trackballs, track points^23^, direct-input pen tablets^50^, and 3D pointing devices with haptic feedback^51^. According to this model, the relationship between the MT required to traverse a straight channel with a specific amplitude (*A*) and width (*W*) is represented by the following expression (Fig. 7: upper image)^22^:

1 $$\begin{aligned} MT = a + b \frac{A}{W} \end{aligned}$$

*a* and *b* are empirically determined constants, while *A*/*W* is also known as the index of difficulty (*ID*). Hence, as the amplitude lengthens or the width narrows, the difficulty of passing through the channel increases, and the MT lengthens. The average cursor speed (*V*) is represented by the following expression^22^:

2 $$\begin{aligned} V = a + b W \end{aligned}$$

This indicates that the speed at which an object passes through a channel depends on the channel width and that it quickens as the width increases and slows as the width narrows.

It has been suggested that the *ID* can be defined not only for a straight channel with a constant channel width but also for various other channel conditions. It has also been suggested that the MT can be predicted by a model based on the *ID*, as shown in the following expression^22^:

3 $$\begin{aligned} MT = a + b \times ID \end{aligned}$$

For example, in a case with a gradually narrowing channel, the *ID* can be defined using the channel widths at the starting and end lines of the channel (see $W_1$ and $W_2$ of Fig. 7: lower image), as in the following expression^22^:

4 $$\begin{aligned} ID = \frac{A}{W_2 -W_1} \ln {\frac{W_2}{W_1} } \end{aligned}$$

In addition, Yamanaka et al. conducted an experiment that included a gradually widening channel with $W_1$ and $W_2$ inverted above the narrowing channel^50^. Their experimental results suggested that the speed of a passing cursor becomes progressively slower in narrowing channels and progressively faster in widening channels; however, model corrections that depend on the orientation of the given channel may allow for more accurate MT predictions.

## Supplementary Information

Supplementary Figures.

## Data Availability

The datasets generated and analyzed during the current study are available from the corresponding author on reasonable request.
