# Supplementary material for: Visual background information modulates motor contagions in humans
Source: Sci Rep. 2024 Aug 13;14:18789. doi: 10.1038/s41598-024-69535-9 (PMC11322384; doi:10.1038/s41598-024-69535-9)
Supplement: Supplementary file 1 — Supplementary Figures. [file 41598_2024_69535_MOESM1_ESM.pdf]

Supplementary Information

Title: Visual background information modulates motor contagions in humans

Authors: Hiroto Saito, Kentaro Fukuchi, Masahiko Inami, and Gowrishankar Ganesh

Supplementary figure

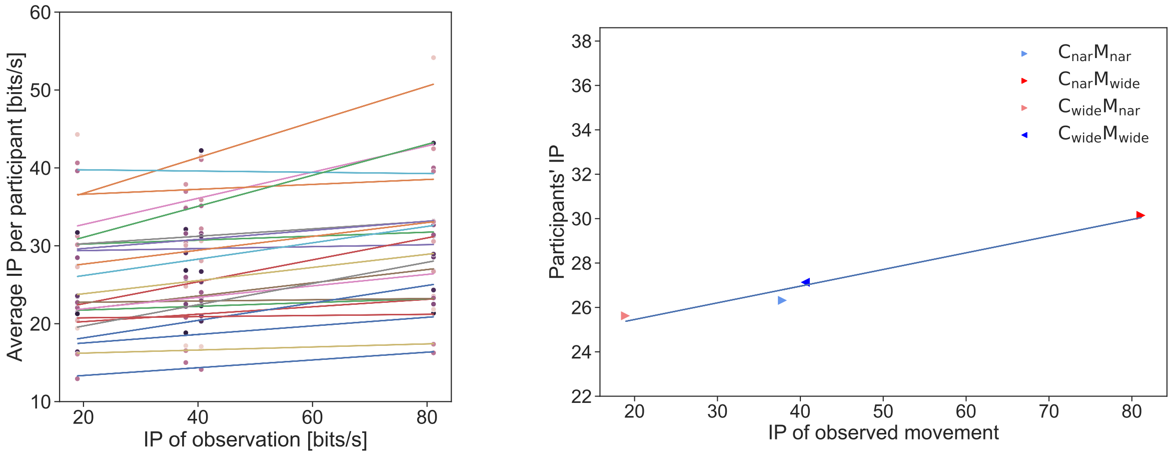

Figure S1. The relationship between the index of performance (IP) of the observed movements and IP of the participant's movements in Experiment-1. The IP is a generalized performance index independent of the path conditions in *Fitts' law* and the *steering law*, as shown in the following expression:

$$IP = ID/MT$$

The graph is a scatterplot with the x-axis as the IP of the observed movement in the observation (*Obs*) block and the y-axis as the IP of the participant in the action (*act*) block after the observation (i.e., blocks 2–5; left: mean value of each participant, right: mean value of all the participants). The straight line represents the regression line. Although the number of points is insufficient to guarantee the results' reliability, the plot of the mean value of all the participants (i.e., right graph) shows a positive correlation between the participants' IP and the IP of the observed movements ($y = 0.075x + 23.954$, $R^2 = 0.973$).

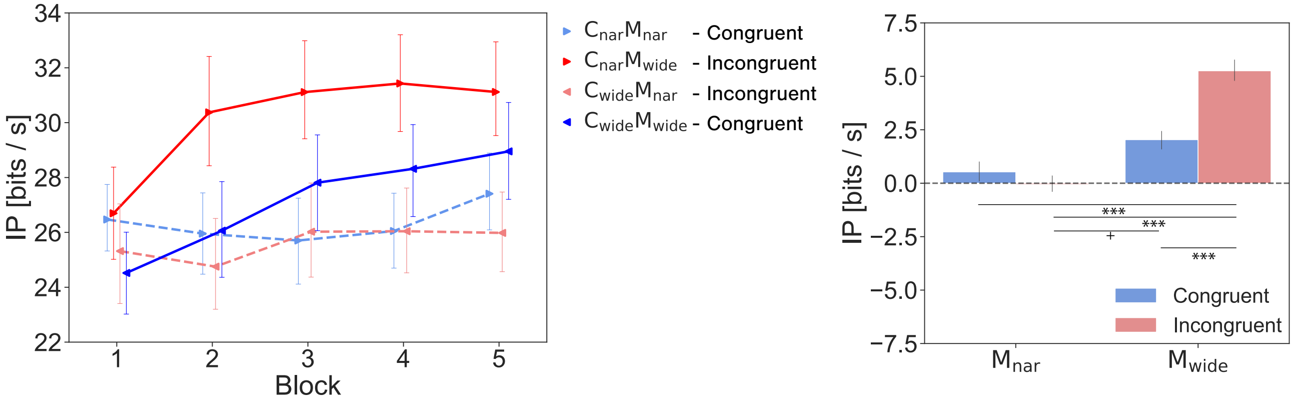

Figure S2. The figure shows the IP in Experiment-1. The left panel shows the changes in the IP of the participants' steering tasks across the *act* blocks in each condition. The right panel shows the difference between the baseline and the average value in blocks 2 to 5 under each condition. The error bars show the standard errors. $^{+}p < .1$, $^{*}p < .05$, $^{**}p < .01$, $^{***}p < .001$. We also analyzed IP as normalized data between channel width in *act* blocks, but the trend of results was generally similar to movement time (MT) as shown in Figure 5, so we described raw MT data in the main manuscript. Normality was rejected for some of the data groups, and the two-way repeated measures ANOVA (2 *prediction error* $\times$ 2 *movements*) with the non-parametric aligned rank transform (ART) procedure showed a significant main effect of *congruency* ($F(1,23) = 6.034$, $p = .022$, $\eta_P^2 = .208$), main effect of *movements* ($F(1,23) = 48.703$, $p < .001$, $\eta_P^2 = .679$), and interaction ($F(1,23) = 10.104$, $p = .004$, $\eta_P^2 = .305$). The post-hoc analysis of interaction, multiple comparisons between the six pairs consisting of four combinations, showed the statistically significant differences in the average IP between $C_{wide}M_{nar}$-$C_{nar}M_{wide}$ ($adj.p < .001$, $C_{wide}M_{nar} > C_{nar}M_{wide}$), $C_{wide}M_{wide}$-$C_{nar}M_{wide}$ ($adj.pp < .001$, $C_{wide}M_{wide} > C_{nar}M_{wide}$), and $C_{nar}M_{nar}$-$C_{nar}M_{wide}$ ($adj.p < .001$, $C_{nar}M_{nar} > C_{nar}M_{wide}$). However, the $C_{wide}M_{nar}$-$C_{wide}M_{wide}$ comparison missed the significant difference, which was observed in the MT ($adj.p = .070$, C_{wide}).

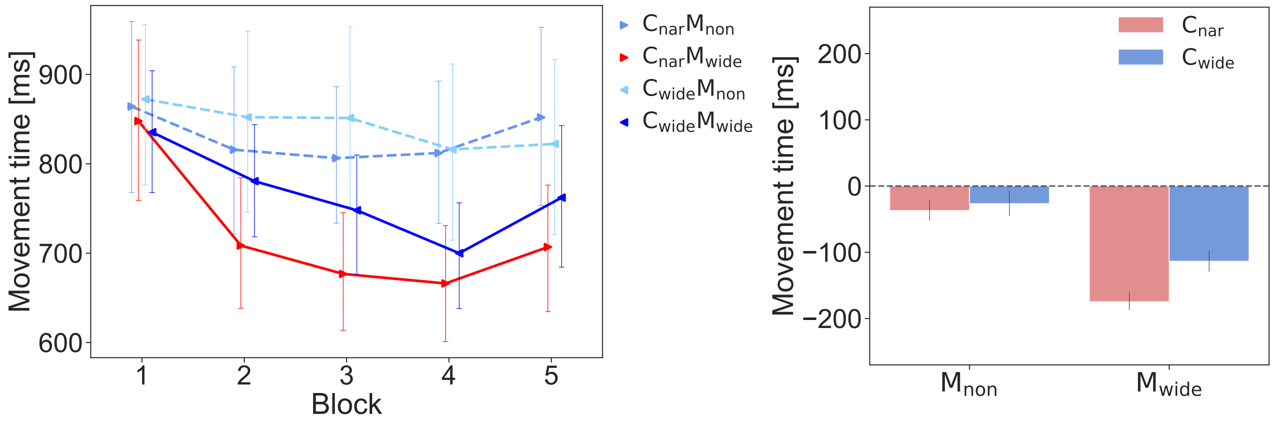

Figure S3. The MT of Experiment-2 obtained with a sample size of twenty. Following a reviewer's suggestion, we also analyzed a total of twenty participants in Experiment-2, adding 8 new participants to the original 12, for a more accurate analysis of the interaction. Specifically, we conducted Experiment-2 with the eight additional participants who did not participate in Experiment 1. The additional participants attended four sessions of two factors (2 *presence of movements* $\times$ 2 *channels*) in random order. This sample size was calculated by G * Power 3.1 using half of the effect size of the channel obtained in Experiment-1 as the predictive effect size ($f = 0.35$). The left panel shows the changes exhibited by the participants' MTs across the *act* blocks under each condition. The right panel shows the differences between the baseline and the average values produced across blocks 2 to 5 under each condition. The error bars show the standard errors. The ANOVA with ART showed only a main effect of *presence of movements* as in the manuscript (*presence of movements*: $F(1,19) = 16.520$, $p < .001$, $\eta_P^2 = .465$; *channels*: $F(1,19) = 2.933$, $p = .203$, $\eta_P^2 = .134$; interaction: $F(1,19) = 2.412$, $p = .137$, $\eta_P^2 = .112$).
